# The Mevalonate Pathway Is Important for Growth, Spore Production, and the Virulence of *Phytophthora sojae*

**DOI:** 10.3389/fmicb.2021.772994

**Published:** 2021-12-22

**Authors:** Xinyu Yang, Xue Jiang, Weiqi Yan, Qifeng Huang, Huiying Sun, Xin Zhang, Zhichao Zhang, Wenwu Ye, Yuanhua Wu, Francine Govers, Yue Liang

**Affiliations:** ^1^College of Plant Protection, Shenyang Agricultural University, Shenyang, China; ^2^Liaoning Key Laboratory of Plant Pathology, Shenyang Agricultural University, Shenyang, China; ^3^Hunan Plant Protection Institute, Hunan Academy of Agricultural Sciences, Changsha, China; ^4^College of Plant Protection, Nanjing Agricultural University, Nanjing, China; ^5^Laboratory of Phytopathology, Wageningen University & Research, Wageningen, Netherlands

**Keywords:** *Phytophthora sojae*, the mevalonate pathway, lovastatin inhibitor, geranylgeranyl diphosphate synthase, spore production, virulence

## Abstract

The mevalonate (MVA) pathway in eukaryotic organisms produces isoprenoids, sterols, ubiquinone, and dolichols. These molecules are vital for diverse cellular functions, ranging from signaling to membrane integrity, and from post-translational modification to energy homeostasis. However, information on the MVA pathway in *Phytophthora* species is limited. In this study, we identified the MVA pathway genes and reconstructed the complete pathway in *Phytophthora sojae in silico*. We characterized the function of the MVA pathway of *P. sojae* by treatment with enzyme inhibitor lovastatin, deletion of the geranylgeranyl diphosphate synthase gene (*PsBTS1*), and transcriptome profiling analysis. The MVA pathway is ubiquitously conserved in *Phytophthora* species. Under lovastatin treatment, mycelial growth, spore production, and virulence of *P. sojae* were inhibited but the zoospore encystment rate increased. Heterozygous mutants of *PsBTS1* showed slow growth, abnormal colony characteristics, and mycelial morphology. Mutants showed decreased numbers of sporangia and oospores as well as reduced virulence. RNA sequencing analysis identified the essential genes in sporangia formation were influenced by the enzyme inhibitor lovastatin. Our findings elucidate the role of the MVA pathway in *P. sojae* and provide new insights into the molecular mechanisms underlying the development, reproduction, and virulence of *P. sojae* and possibly other oomycetes. Our results also provide potential chemical targets for management of plant *Phytophthora* diseases.

## Introduction

Oomycetes are a diverse group of fungus-like eukaryotic microorganisms that are phylogenetically distinct from fungi and classified within the stramenopila kingdom. Various types of reproductive spores are produced in the oomycete life cycle, asexual sporangia, zoospores, and sexual oospores, and pathogenic oomycetes rely on both structures to achieve infection (Judelson and Blanco, 2005). There are more than 100 species of oomycetes that cause destructive diseases in agriculture, and they are considered the most destructive species of plant pathogens (Kroon et al., 2012; Kamoun et al., 2015). For example, *Phytophthora infestans* is a notorious oomycete pathogen that causes late blight in potato and tomato worldwide (Fry, 2008). *Phytophthora sojae* is another notorious species that causes root and stem rot disease in soybean, resulting in approximately $2 billion losses worldwide annually (Tyler, 2007). In addition, the extraordinary genetic plasticity of *Phytophthora* enables the pathogens to adapt rapidly to, and overcome, chemical or host resistance, which makes controlling the diseases more difficult (Fawke et al., 2015).

**Figure 1:**
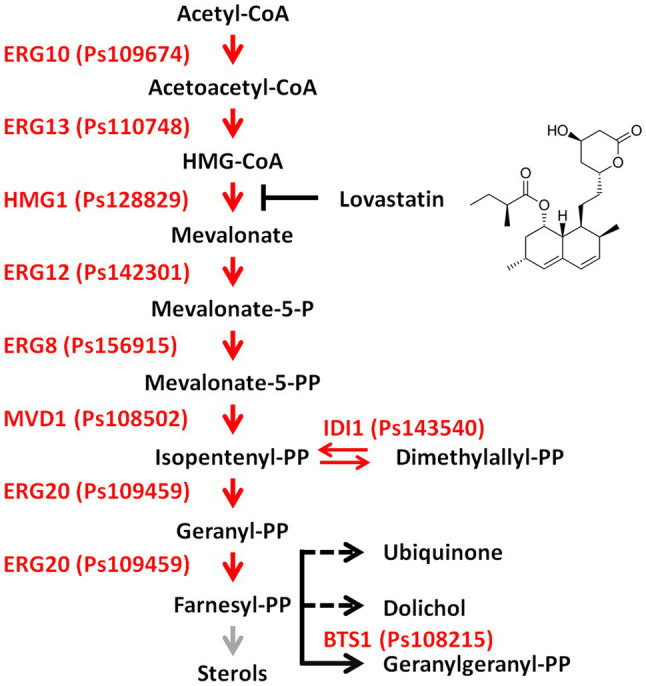
Overview of the mevalonate (MVA) pathway in *Phytophthora sojae*. Enzymes and the corresponding gene ID of the MVA pathway in *P. sojae* are shown beside each arrow head. The main trunk of the MVA pathway is indicated by red arrows. The grey arrow indicated the deficient sterol biosynthesis sub-branch in *Phytophthora* species. The black arrow indicated the GGPP biosynthesis sub-branch, while the dashed arrow indicated that the sub-branches were not reported in *Phytophthora* species. The enzyme inhibitor lovastatin and chemical structure are also indicated on the right of the pathway. Acetyl-CoA, acetyl coenzyme A; Aceto-acetyl-CoA, aceto-acetyl coenzyme A; HMG-CoA, 3-hydroxy-3-methylglutaryl coenzyme A; Mevalonate-5-P, mevalonate phosphate; Mevalonate-5-PP, mevalonate diphosphate; Isopentenyl-PP, isopentenyl diphosphate; Dimethylallyl-PP, dimethylallyl diphosphate; Geranyl-PP, geranyl diphosphate; Farnesyl-PP, farnesyl diphosphate; and Geranylgeranyl-PP, geranylgeranyl diphosphate.

Isoprenoids are the most diverse secondary metabolites in eukaryotes. They are produced *via* two independent pathways, the mevalonate (MVA) pathway which is present in most organisms, and the 2-C-methyl-D-erythritol 4-phosphate (MEP) pathway which is specific for plants and green algae (Vranova et al., 2013). In the initial steps of the MVA pathway, acetyl-CoA is converted into isopentenyl diphosphate (IPP) and dimethylallyl diphosphate (DMAPP), then IPP and DMAPP condense to produce geranyl diphosphate and farnesyl diphosphate (FPP; Figure 1; Nes, 2011, Fabris et al., 2014). FPP feeds into different sub-branches of the MVA pathway as a universal precursor (Figure 1; Hemmi et al., 2003, Fabris et al., 2014). Among the many enzymes participating in the MVA pathway, 3-hydroxy-3-methylglutaryl-CoA reductase (HMG-CoA reductase, HMGR) is of particularly interest (Figure 1). This enzyme functions in the rate-limiting catalytic step and is the target of statins, the widely used cholesterol-lowering drugs (Goldstein and Brown, 1990; Lorenz and Parks, 1990). Binding of statins, such as lovastatin, to HMGR can alter the enzyme conformation, leading to decreased sterol biosynthesis (Istvan and Deisenhofer, 2001; Stancu and Sima, 2001). The ability of statins to inhibit sterol biosynthesis has been verified in human pathogenic fungi and provides antifungal properties (Westermeyer and Macreadie, 2007; Liu et al., 2009; Bellanger et al., 2016).

The sterol biosynthesis sub-branch of the MVA pathway has been identified in animals, fungi, and land plants (Figure 1; Desmond and Gribaldo, 2009; Ruiz-Sola et al., 2016). In contrast, oomycete species in the genera *Phytophthora* and *Pythium* are sterol auxotrophs that utilize exogenous sterols from the environment or from host plants to support their growth and development (Marshall et al., 2001; Yousef et al., 2009). Genome analyses revealed that most enzymes of the sterol biosynthesis sub-branch are absent in *Phytophthora* species, which makes them unable to synthesize sterol independently (Tyler et al., 2006; Dahlin et al., 2017). However, two enzymes (ERG3 and DHCR7) that function in the last steps of sterol biosynthesis were reported to present in *Phytophthora* and *Pythium* species (Wang et al., 2021a). These two enzymes were proposed to modify the exogenous sterols, especially DCHR7 was effective in converting ergosterol into brassicasterol in *Phytophthora capsici* (Wang et al., 2021b). Conversely, *Saprolegnia parasitica* and *Aphanomyces euteiches* are sterol autotrophs (i.e., synthesis cholesterol derivatives and fucosterol, respectively) and the sterol biosynthesis sub-branch has been identified in these two oomycete pathogens (Madoui et al., 2009; Warrilow et al., 2014). Moreover, genome and transcriptome analyses revealed that certain MVA pathway genes were potentially present in some *Phytophthora* species and the branch point enzyme geranylgeranyl diphosphate synthase (GGPS) in the terpenoid biosynthesis sub-branch of the MVA pathway was identified (Dahlin et al., 2017; Lerksuthirat et al., 2017).

GGPS utilizes FPP as precursor for the production of geranylgeranyl diphosphate (GGPP), the first product in terpenoid biosynthesis sub-branch (Figure 1; Hemmi et al., 2003; Beck et al., 2013). GGPS contains three highly conserved motifs: the G(Q/E) motif and two aspartate-rich motifs, designated the first aspartate-rich motif (FARM) and the second aspartate-rich motif (SARM; Hemmi et al., 2003). Based on the features of these conserved motifs, GGPSs are classified into three types: type I GGPS found in archaebacteria, type II GGPS found in bacteria and plants, and type III GGPS found in animals and fungi (Hemmi et al., 2003). In plants, GGPP serves as the precursor for photosynthetic pigments (carotenoids and chlorophylls) and phytohormones [gibberellins (GAs), abscisic acid, and strigolactone] (Thabet et al., 2012; Ruiz-Sola et al., 2016). For example, the 10 functional GGPS genes identified in the *Arabidopsis* genome have differential spatiotemporal expression and subcellular localization (Okada et al., 2000; Beck et al., 2013). Among these GGPSs, mutation in GGPS1 affected chloroplast development while deletion of GGPS11 resulted in developmental defects in *Arabidopsis* (Ruppel et al., 2013; Ruiz-Sola et al., 2016). Overexpression of a sunflower GGPS gene in tobacco led to improved GA levels, and thereby an enhanced growth rate, early flowering, and increased seed yield (Tata et al., 2016).

In fungi, GGPS is not only essential for the production of isoprenoid-derived substances, such as antibiotics, the antitumor agent Taxol, and the antimalarial agent artemisinin, but also crucial for fungal development and virulence (Saikia and Scott, 2009; Singkaravanit et al., 2010a). For example, the *Saccharomyces cerevisiae* genome carries one GGPS gene, *BTS1*, that when deleted caused differential growth rates at lower temperatures (Jiang et al., 1995). The two different types of GGPS (*ggs1* and *ggs2*) isolated from filamentous fungi are responsible for production of GGPP or secondary metabolites (GA or paxilline), but the biological function has not been reported (Singkaravanit et al., 2010a). In the entomopathogenic fungus *Metarhizium anisopliae*, *ggs1* is constitutively expressed throughout fungal growth and is responsible for GGPP production (Singkaravanit et al., 2010b). Disruption of *ggs2* results in abolished helvolic acid production, delayed sporulation, and decreased toxicity to host insects (Singkaravanit et al., 2010a).

In *Phytophthora* spp., the MVA pathway has not been investigated and the function of the MVA pathway in development is not clear. In this study, we reconstructed the MVA pathway in *P. sojae* by identifying the whole set of genes and studied the effects of inhibiting the rate-limiting enzyme HMGR by lovastatin treatment. We also generated GGPS-deficient *P. sojae* mutants by CRISPR/Cas9-mediated deletion of the GGPS encoding gene *PsBST1*, analyzed their phenotypes, and compared the transcriptomes of wild-type (WT) *P. sojae* and a lovastatin-treated sample. This study shows that the MVA pathway plays essential roles in vegetative growth, development, reproduction, and virulence in *P. sojae* and uncovers a potential chemical target for sustainable management of soybean root rot caused by *P. sojae* and other *Phytophthora* diseases.

## Materials and Methods

### Oomycete Strains and Culture Conditions

Wild-type oomycete strains *P. sojae* P6497, *P. infestans* T30-4, *P. capsici* LT263, and *Pythium ultimum* F18-6 were generously provided by Yuanchao Wang (Nanjing Agricultural University, Nanjing, China). All strains and *PsBTS1* deletion mutants were maintained on 10% V8 agar slants at 14°C in the dark at College of Plant Protection, Shenyang Agricultural University.

### Gene Mining and Phylogenetic Analysis

The deduced amino acid sequences for genes related to the MVA pathway were downloaded from the yeast *S. cerevisiae* genome database^1^ and used as BlastP queries to search for homologues from *P. sojae* (Joint Genome Institute, JGI). The homologues of *P. infestans*, *S. parasitica*, and *Py. ultimum* were searched from the Ensembl Genome database. The homologues of *P. capsici* were searched from NCBI GenBank. Each candidate amino acid sequence in *P. sojae* was blasted against the UniProt database to predict its potential functions. Based on a database derived from a global transcriptome investigation of *P. sojae* by 3′-tag gene expression analysis (Ye et al., 2011), the expression data of the nine genes in the MVA pathway were retrieved and transformed with log2 in this study. An expression heatmap was generated using Multiple Experiment Viewer with the hierarchical clustering method. GGPS proteins from different organisms were also downloaded from the National Center for Biotechnology Information (NCBI) database, and a phylogenetic tree was generated in MEGA 6.0 with the neighbor-joining method. The GGPS protein sequences were aligned using BioEdit software.

### Inhibition Assay Under Statin Treatment

Lovastatin was purchased from Solarbio (Beijing, China) and a 20 mg/ml stock solution was prepared and stored at −20°C. The mycelial inhibition assays were preliminarily performed on V8 medium supplemented with different concentrations of lovastatin. Briefly, *P. sojae* was grown on V8 medium for 5 days; eight 5-mm mycelium plugs were transferred to a new plate; the plugs were flooded with 5 ml water containing various concentrations (0, 20, 40, and 80 μg/ml) of lovastatin; flooding was repeated every 40 min for 4 h; and then, plugs were incubated for another 5 h until sporangia formation. Zoospore suspensions (200 μl, 1 × 10^6^ spores/ml) were gently mixed with lovastatin solution to final concentrations of 0, 20, 40, and 80 μg/ml, dropped into a glass slide, and incubated at 25°C in the dark for 40 min. The numbers of zoospores and encysted zoospores in a 10-μl suspension were counted; counts were repeated for a total of five times. After growth for 30 days on V8 medium supplied with the different concentrations of lovastatin, a 5-mm mycelium plug adjacent to the inoculation site was excised and the number of oospores was counted under a microscope (AXIO Scope A1; Zeiss, Oberkochen, Germany).

### Plasmid Construction and CRISPR/Cas9-Mediated Gene Editing in *P. sojae*

We used two single guide RNAs (sgRNAs) and the HDR gene replacement method to delete *PsBTS1* (Fang and Tyler, 2016). We designed two efficient sgRNA sequences (sgRNA1-GCGTACATCAGCGAGCTGCC and sgRNA2-TCCAGAAGATGTCCAGCCCC) and inserted them to the pYF515 plasmid according to the method described by Fang et al. (2017). The 1,000-bp flanking sequences were ligated to the fluorescence gene eGFP (760 bp) on each side, which served as the donor DNA. The recombinant donor DNA sequences were inserted into pBlueScript SK II+ plasmid. All plasmids used in the CRISPR/Cas9 system were constructed as described by Fang et al. (2017). The full coding sequence of *PsBTS1* was amplified from the cDNA template and inserted into pTOReGFP for subcellular localization. All the plasmids were sequenced at the Beijing Genomics Institute (BGI, Shenzhen, China) before use. The CRISPR/Cas9-mediated gene replacement strategy and PEG-mediated protoplast transformation in *P. sojae* were used to obtain *PsBTS1* deletion mutants followed by the protocol presented by Fang and Tyler (2016). The gene deletion mutants were verified by PCR with genomic DNA to further confirm target gene deficiency; four individual deletion mutants from independent transformation were obtained and used as biological replicates for genotypic analysis subsequently. The PsBTS1 undeleted transformants were used as the control strains (CK) in the subsequent analyses.

### Yeast Strains, Culture Conditions, and Transformation

The yeast gene deletion mutant strain BY4741 *bts1Δ* (*YPL069C*) and the WT strain BY4741 (*MATa his3Δ1*, *leu2Δ0 met15Δ0 ura3Δ0*) were purchased from Dharmacon (Cambridge, United Kingdom) and preserved in 30% glycerol. The full-length *PsBTS1* sequence was cloned into pYES2 (Invitrogen, Carlsbad, CA, United States) and transformed into the BY4741 *bts1Δ* mutant strain using a Yeastmaker Yeast Transformation System 2 kit (Takara, Dalian, China) following the manufacturer’s protocol. The empty plasmid pYES2 was transformed into yeast WT strain BY4741 as well as the *bts1Δ* mutant, which served as the controls. Colonies grew on Synthetic Defined medium lacking uracil containing glucose were further screened by PCR. The transformed yeast cells were cultured overnight in YPD broth, then rinsed with water to remove YPD completely. The yeast cell solution was adjusted to an optical density at 600 nm of 0.2, then serially diluted 10 times. A 5-μl drop from each dilution was spotted into YPD medium without uracil, containing either glucose or galactose, and grown at 14, 25, or 30°C for 7 days.

### Subcellular Localization by GFP Visualization

*Phytophthora sojae* transformants expressing PsBTS1-GFP fusion proteins and GFP were individually subcultured twice on V8 agar medium. Four mycelium plugs were transferred to a new plate, immersed in 10% V8 broth, and cultured for 2 days to obtain mycelia. The mycelia were rinsed twice with sterile distilled water to perform fluorescence microcopy with a confocal laser scanning microscope (FV3000; Olympus, Tokyo, Japan) using excitation/emission wavelengths of 488/515 nm.

### Phenotype Characterization of *PsBTS1* Gene Deletion Mutants

All strains were subcultured twice on V8 agar before characterization analysis. The growth assay was carried out on Plich’s medium using fresh cultures started on V8 agar and grown for 3–5 days at 25°C in the dark. Before the colony reached the edge of the plate, a mycelium plug 5 mm in diameter was taken and inoculated on Plich’s medium. The plates were incubated at 25°C in the dark, then photographed after 10 days of incubation, and the mycelial growth and distance between branches were measured. The sporangia formation of *PsBTS1* mutants was induced by repeatedly flooding mycelial plugs with sterile water, the oospores numbers of *PsBTS1* mutants on a 5-mm mycelium plug were determined by visual counting under microscope.

### Virulence Assays

The susceptible soybean cultivar Hefeng 47 was grown in vermiculite at 25°C for 4 days in the dark; the etiolated soybean seedlings were used to assess the virulence of *P. sojae* strains. Zoospore preparation was performed as described by Hua et al. (2008). Zoospore suspensions (5 μl, 20 zoospores/μl) of *PsBTS1* mutants were inoculated directly into hypocotyls of soybean seedlings, or zoospore suspensions were gently mixed with lovastatin solution to final lovastatin concentrations of 0, 20, 40, and 80 μg/ml and then inoculated on hypocotyls of soybean seedlings. The inoculated etiolated soybean seedlings were maintained at 25°C in the dark. The soybean seedlings were photographed and evaluated at 48 hpi. The epidermal cells from inoculation sites were excised at 12 and 24 hpi to explore the impeded virulence. All assays were performed three times.

### Transcriptional Analysis of Lovastatin-Treated *P. sojae* Sample

To better understand the effects of MVA pathway on gene expression, RNA-seq was conducted using BGISEQ-500 sequencing platform at BGI. Based on our results, lovastatin (80 μg/ml)- and water-treated *P. sojae* strains were used for RNA-seq with three biological replicates per treatment. Trimmomatic was used to remove adaptors and low-quality reads from the raw data (Bolger et al., 2014); SOAP2 (Li et al., 2009) was used to map the filtered clean reads to the reference genome (*Phytophthora sojae* genome 1.0). The transcriptome data were assembled using StringTie (Pertea et al., 2016) to generate new transcripts, from which gene expression was calculated as the fragments per kilobase per million mapped reads. Differential expression analysis was completed using DESeq2 (Anders and Huber, 2010), where a fold change (treated expression/untreated expression) ≥2 and value of *p* ≤ 0.05 were considered to indicate differential expression. Scatter plots were generated using the R base plot function. The enrichment analysis of GO terms and KEGG pathways from the JGI^2^ was accomplished using the clusterProfiler package in R (Yu et al., 2012) with the hypergeometric distribution test and corrected value of *p* to enrich significant gene functions and pathways.

### RNA Extraction and qRT-PCR Gene Expression Analysis

The *P. sojae* WT strain and a representative *PsBTS1* deletion mutant (T4) were grown on 10% V8 agar plates for 3 days at 25°C in the dark. Five mycelia plugs from the edges of colonies were transferred to new plates and cultured in 10% V8 broth. The WT strain was treated with lovastatin (80 μg/ml). The mycelia were collected after 4 days of growth at 25°C in the dark for RNA extraction. RNA was extracted using a PureLink RNA Mini Kit (Thermo Fisher, Shanghai, China) and the quality was determined by 1.0% agarose gel electrophoresis. Total RNA (1–2 μg) was used to synthesize the first cDNA strand with oligo(dT) primers and an M-MLV Reverse Transcriptase Kit (Thermo Fisher, Shanghai, China). The primers for qRT-PCR were designed using Primer3 with an amplicon size range of 150–200 bp. SYBR green qRT-PCR assays were performed on an ABI StepOne system (Thermo Fisher, Foster City, United States) to evaluate expression levels of selected genes. Relative gene expression was calculated using the conserved *actinA* (accession number Ps108986) levels as internal controls (Hua et al., 2013; Yang et al., 2013). Means and SDs were calculated using data from three replicates. All assays were carried out following the manufacturers’ protocols.

### Statistical Analysis

All of the data were analyzed for statistical significance (*p* < 0.05) using GLM procedure for ANOVA or the Student’s *t* test with SAS software (version 9.1; SAS Institute Inc., Cary, NC, United States).

## Results

### The HMGR Inhibitor Lovastatin Reduces Growth, Zoosporulation, and Virulence of *P. sojae*

**Figure 2:**
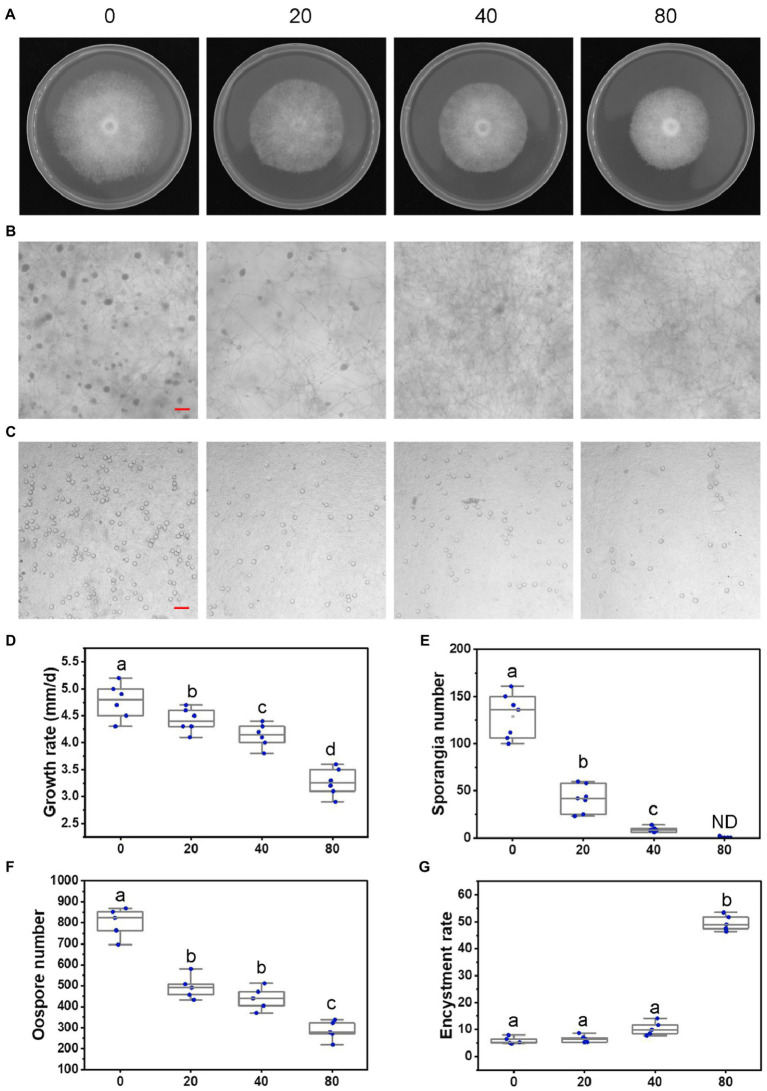
Lovastatin suppresses growth, sporangia, and oospore formation and stimulates zoospore cysts in *P. sojae*. **(A)** *P. sojae* grown without and with lovastatin. **(B,C)** Microscopic visualization of sporangia in 15 days old cultures **(B)** and oospores in 30 days old cultures **(C)** grown without and with lovastatin. **(D)** *P. sojae* growth rates under treatment with various concentrations of lovastatin. **(E)** Sporangia counts on a 5-mm-diameter agar plug, ND, not detected. **(F)** Oospore counts on a 5-mm-diameter agar plug. **(G)** Encystment rates of zoospores harvested from 15 days old cultures grown without and with lovastatin. Lovastatin concentrations: 0, 20, 40, and 80 μg/ml; a, b, c, and d indicate significant differences (*p* < 0.05). Bar: 100 μm.

To elucidate the role of the MVA pathway, *P. sojae* was treated with lovastatin, a specific inhibitor of the rate-limiting enzyme HMGR (Figure 1). In *S. cerevisiae*, 10 μg/ml is sufficient to block the MVA pathway (Lorenz and Parks, 1990). In *P. sojae*, we observed that the growth rate decreased with increasing concentrations of lovastatin (Figures 2A,D) and the highest concentration that was tested, i.e., 80 μg/ml, mycelial growth was reduced with 31.9%. Similar inhibitory effects of lovastatin were observed in *P. infestans*, *P. capsici*, and *Py. ultimum* (Supplementary Figure S1). These results indicate that interruption of the MVA pathway by lovastatin treatment impairs the vegetative growth of *P. sojae* and closely related oomycetes.

To further assess the effects of the MVA pathway on the asexual and sexual development of *P. sojae*, sporangia and oospore formation were quantified in cultures grown in the presence of increasing concentrations of lovastatin (Figures 2B,C). Lovastatin treatment significantly inhibited sporangia and oospore formation (Figures 2E,F). Specifically, sporangia numbers were reduced by 67.7 and 93.3% at lovastatin concentrations of 20 and 40 μg/ml, respectively, while no sporangia were observed at 80 μg/ml (Figures 2B,E). Oospore numbers decreased by 38.4, 46.2, and 64.5%, respectively (Figures 2C,F). Lovastatin treatment also increased the zoospore encystment rate by 8.4-fold (Figure 2G).

**Figure 3:**
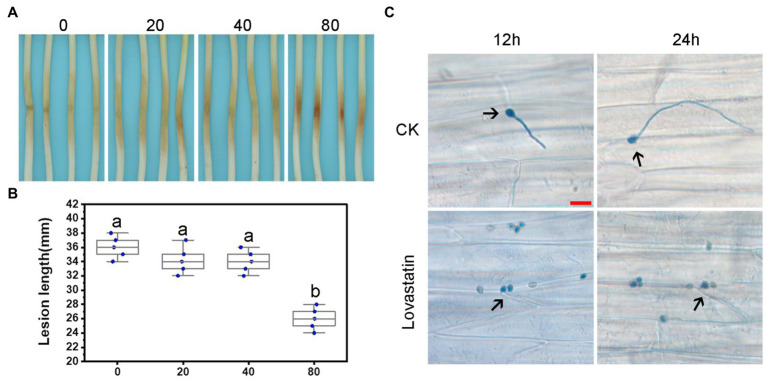
Lovastatin treatment led to reduced virulence of *P. sojae*. Zoospore suspensions pretreated with various concentrations (0, 20, 40, and 80 μg/ml) of lovastatin were inoculated into hypocotyls of soybean cultivar Hefeng 47. **(A)** Photos and **(B)** lesion lengths were taken at 48 hpi. **(C)** The germinated cysts of untreated zoospores (CK) were observed on soybean epidermal cells at 12 and 24 hpi; lovastatin (80 μg/ml)-treated zoospores had not germinated at 12 or 24 hpi; and a and b indicate significant differences (*p* < 0.05). Bar: 25 μm.

In order to assess the effect of lovastatin on the virulence of *P. sojae*, zoospores were treated with different concentrations of lovastatin and subsequently used for inoculation of soybean hypocotyls. At 48 h post-inoculation (hpi), the length of lesions caused by zoospores treated with 80 μg/ml lovastatin decreased by 26.6% compared to mock-treated zoospores (Figures 3A,B). At 12 and 24 hpi, the zoospores without lovastatin treatment were able to germinate and penetrate epidermal cells, whereas the lovastatin-treated zoospores were unable to germinate (Figure 3C). The multiple effects of lovastatin, such as increased zoospore encystment rate, inhibited zoospores germination on host surface and mycelial extension, suggested that lovastatin reduces the virulence of *P. sojae* in this study.

### Mining the *P. sojae* Genome for Genes Involved in the MVA Pathway

The deduced amino acid sequences of genes involved in the MVA pathway were downloaded from the *S. cerevisiae* genome database and used as BlastP queries to mine for potential homologues in the *P. sojae* genome. In total, nine homologues that could construct a complete MVA pathway were found (Figure 1; Supplementary Table S1). These genes showed pronounced similarity to homologues in *S. cerevisiae*, ranging from 29.5 to 51.4% at the amino acid level. In *S. cerevisiae*, most enzymes involved in the catalytic steps of the MVA pathway are encoded by a single gene, but the catalyzing enzyme HMGR is encoded by a pair of genes (*HMG1*/*HMG2*). In *P. sojae*, all homologues in the MVA pathway were identified, but only one HMGR gene was present (Supplementary Table S1). Furthermore, the *P. sojae* genes also showed high similarity to the identified homologues in the MVA pathways of *S. parasitica*, *P. infestans* (Dahlin et al., 2017), and *P. capsici* and *Py. ultimum*, with protein sequence identities in the range of 41.5–70.8, 73.3–93.9, 70.2–93.9, and 49.6–84.0%, respectively (Supplementary Table S2). The MVA pathway genes in *P. sojae* were further predicted in UniProt, the most similar homologues were identified in other *Phytophthora* spp. The similarity of MVA pathway genes of *P. sojae* with best-hit homologues ranges from 75.4 to 94.5% at the protein level.

Expression analysis of the genes in MVA pathway was conducted at the different development and infection stages. Then, a clustered heatmap was performed and gene expression was clustered into three differential patterns (Supplementary Figure S2). Specially, three genes (Ps143540, Ps142301, and Ps110748) showed relatively low expression levels while two (Ps109674 and Ps128829) were expressed in most stages of development and infection. The remaining four genes showed dynamically differential expression during the different stages, in which the gene encoding the branch point enzyme GGPS (accession number Ps108215; *PsBTS1*) exhibited a more dynamic expression pattern than the other three genes, including the relatively higher expressed in mycelial and infection stages and the lower in sporangia, zoospores, cysts, and germinated cysts. Based on the expression patterns, we hypothesized that GGPS (especially *PsBTS1*) is involved in growth and infection processes of *P. sojae*.

### Characterization of the *P. sojae* GGPP Synthase *PsBTS1*

**Figure 4:**
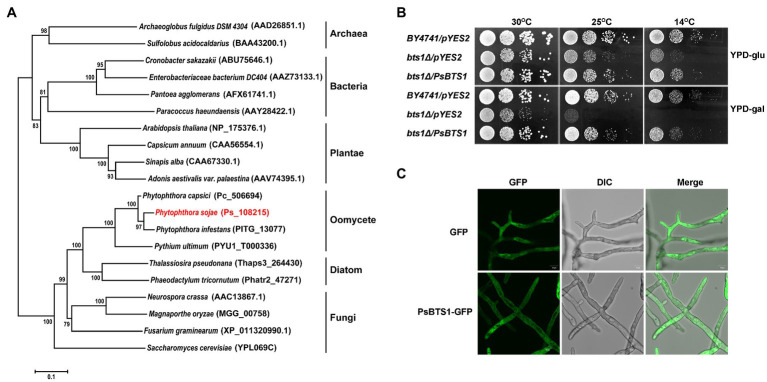
Phylogenetic analysis, enzymatic activity, and subcellular localization of PsBTS1. **(A)** Phylogenetic analysis of GGPSs from different organisms. GGPS proteins from archaea, bacteria, plants, fungi, diatoms, and oomycetes were analyzed using MEGA6; their sequence IDs in the NCBI GenBank or genome databases are included. **(B)** PsBTS1 complements the *Saccharomyces cerevisiae bts1Δ* mutant growth defect at 14 and 25°C. Growth of *S. cerevisiae* strains *BY4741/pYES2*, *bts1Δ*/*pYES2*, and *bts1Δ*/*PsBTS1* was tested on YPD plates with glucose (top panel) and galactose (bottom panel) at 14, 25, and 30°C. **(C)** Subcellular localization of PsBTS1. GFP and PsBTS1-GFP fusion protein were expressed in *P. sojae* and mycelial were analyzed by fluorescence (GFP, 488/515 nm) and bright field (DIC) microscopy. Bar: 10 μm.

The gene encoding GGPP synthase is named *PsBTS1* after its homologue in *S. cerevisiae*. The genome sequence of *PsBTS1* comprises two introns, with a length of 108 and 107 bp, respectively. The 888 bp coding sequence (CDS) of *PsBTS1* encodes a protein of 295 amino acids in length. A phylogenetic tree based on PsBTS1 and its homologues from archaebacteria, bacteria, fungi, plants, other oomycete species, and diatoms is comprised of three main clades, with archaebacteria clustering in one clade, bacteria and plants in another clade, and fungi, oomycetes, and diatoms together in the third clade (Figure 4A). The GGPS protein sequences of selected organisms were further used to identify the conserved motifs. The alignment revealed two aspartic-acid-rich motifs (FARM/SARM) and conserved G(Q/E) motifs (Supplementary Figure S3). Therefore, the GGPSs derived from oomycetes were categorized as type III, together with fungi and diatoms, especially as the FARM and SARM motifs differed from those of plants (Supplementary Figure S3).

### Enzymatic Activity and Subcellular Localization of PsBTS1

To determine if *PsBTS1* encodes an active enzyme, we made use of *S. cerevisiae* cells in which the BTS1 gene (SGD:S000005990) is deleted. This temperature sensitive *bts1Δ* cells do not grow on galactose-containing medium at the non-permissive temperature. We transformed the expression plasmid pYES2 and the same plasmid carrying the CDS of *PsBTS1* into *bts1Δ* yeast cells and tested their growth on glucose-containing (suppression) or galactose-containing (induction) yeast extract–peptone–dextrose (YPD) medium at 14, 25, and 30°C (Figure 4B). All strains grew normally on both glucose- and galactose-containing YPD medium at the regular cultivation temperature of 30°C. However, the WT yeast strain BY4741 grew faster than the *bts1Δ*/pYES2 and *bts1Δ*/*PsBTS1* strains on glucose-containing medium at lower temperatures (14 and 25°C; Figure 4B). On galactose-containing YPD medium, the *bts1Δ*/*PsBTS1* strains formed colonies similar to the WT strains while the growth of *bts1Δ*/pYES2 strains was significantly inhibited (Figure 4B). Since these results demonstrate that *PsBTS1* can complement the growth defect of the yeast *bts1Δ* mutant, we conclude that PsBTS1 has the anticipated enzymatic activity and is thus a GGPP synthase.

To reveal the subcellular localization of PsBTS1, the CDS of *PsBTS1* was fused with green fluorescent protein (GFP) at the N-terminus, and the resultant fusion protein was expressed in *P. sojae*. A *P. sojae* strain with GFP expression was used as a control. The PsBTS1-GFP fusion protein and the control were driven by the constitutive ham34 promoter, which were highly expressed in *P. sojae* with the visual detection of strong green fluorescence. PsBTS1-GFP fusion protein and the control were found to be distributed in the cytoplasm (Figure 4C). In addition, the GFP signal observed in specific localization was not indicated in the magnification of either the PsBTS1-GFP mycelia and the control (Supplementary Figure S4), which suggested the expression of PsBTS1 was in the cytoplasm.

### Phenotype Changes Caused by *PsBTS1* Deletion in the MVA Pathway

**Figure 5:**
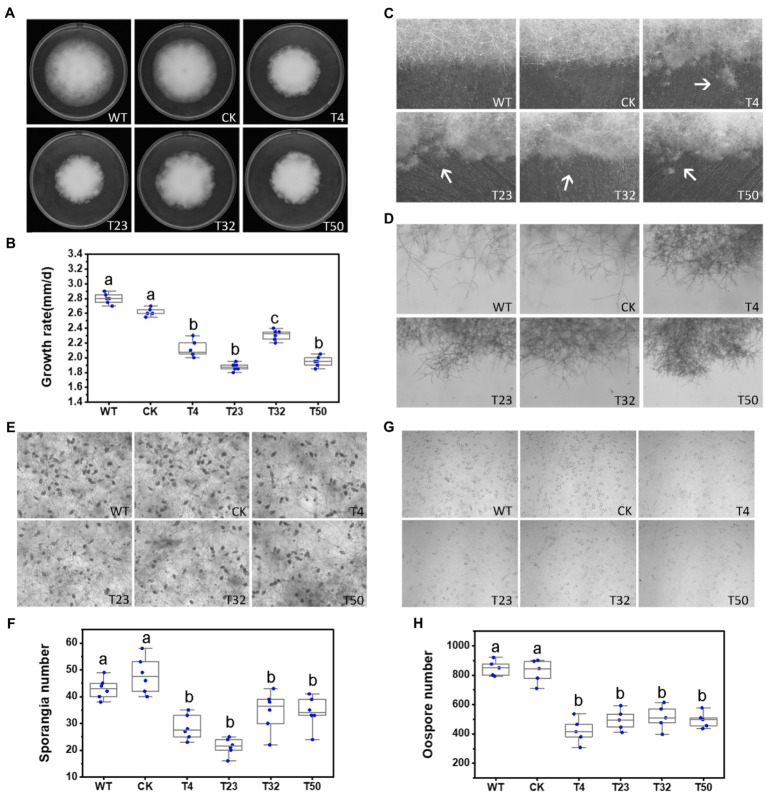
Phenotypic analysis of *PsBTS1* deletion mutants. Comparison of wild-type (WT) *P. sojae* with a control transformant (CK) and four *PsBTS1* deletion mutants (T4, T23, T32, and T50). **(A)** Colony morphology and **(B)** growth rate on Plich medium. **(C)** Colony edges. **(D)** Mycelial morphology of satellite colonies. **(E)** Microscopic visualization and **(F)** quantification of sporangia. **(G)** Microscopic visualization and **(H)** quantification of oospores. In **(F)** and **(H)** a, b, and c indicate significant differences (*p* < 0.05). Bar: 100 μm.

To investigate the role of *PsBTS1* in growth, reproduction, and virulence of *P. sojae*, we disrupted *PsBTS1* in *P. sojae* based on the CRISPR/Cas9-mediated homology-directed repair (HDR) method and replaced it with an exogenous GFP sequence. In this study, four deletion mutants (named T4, T23, T32, and T50) were obtained as the independent biological replicates and verified by PCR and quantitative reverse-transcription PCR (qRT-PCR) expression analyses (Supplementary Figures S5B,C). Editing of *PsBTS1* was observed based on the results of the junction PCR, while the spanning PCR and the internal PCR indicated an allele was replaced by exogenous GFP sequence (Supplementary Figure S5B). While the sequence of undeleted allele of PsBTS1 in the mutants was identical to that of WT (data not shown). All the mutants were heterozygous and showed decreased levels of *PsBTS1* transcript; no homozygous mutants were obtained also by making subcultures of single spores. The expression levels of two neighbor genes (Ps128815 and Ps128817) adjacent to *PsBTS1* were also estimated to avoid potential influence on the expression caused by target gene *PsPTS1* editing. Results indicated that the expression levels of the neighbor genes were similar among the *PsBTS1* deletion mutants and the WT strain (Supplementary Figure S5D). Morphological features of *PsBTS1* mutants were examined and compared to the WT and control (CK) strains at different developmental stages (Figure 5). Compared to WT and CK strains, the mutants exhibited abnormal and smaller colonies (Figure 5A). Specifically, the average rate of mycelial growth decreased by 24.1% (Figure 5B). Meanwhile, *PsBTS1* mutants exhibited rough and irregular colony edges where the aerial mycelial tips attached to the surface of the growth medium and formed individual small satellite colonies (Figure 5C). The newly generated mycelia of *PsBTS1* mutants showed a more condensed hyphal density (Figure 5D), while the distance between branches was shorter in satellite colonies compared to WT and CK strains (Supplementary Figure S6). These results show that deletion of *PsBTS1* results in impaired colony morphology and vegetative growth of *P. sojae*.

**Figure 6:**
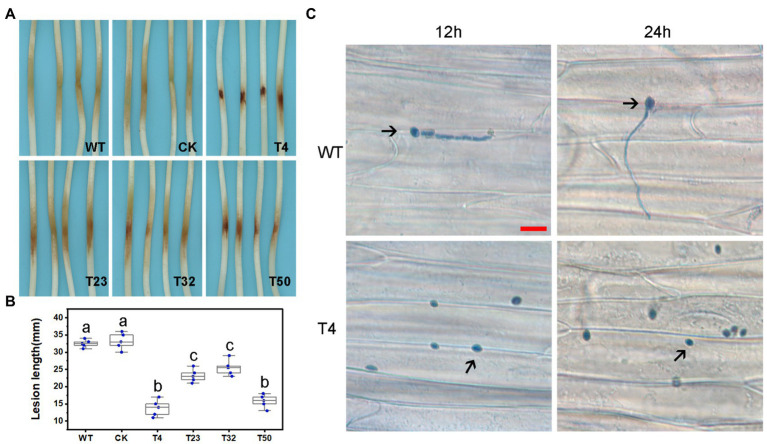
PsBTS1 mutants showed reduced virulence. **(A)** Lesions and **(B)** lesion lengths on hypocotyls of etiolated soybean seedlings. Comparison of WT *P. sojae* with a CK and four PsBTS1 deletion mutants (T4, T23, T32, and T50), a, b, and c indicate significant differences (*p* < 0.05). **(C)** Microscopical examination of the epidermal cell surface at 12 and 24 hpi after inoculation with zoospores from WT *P. sojae* and *PsBTS1* KO T4. Bar: 25 μm.

We also assessed the functions of *PsBTS1* during asexual and sexual development by evaluating sporangia formation, zoospore encystment, and oospore production. The number of sporangia produced by *PsBTS1* mutants decreased compared to the WT and CK (Figures 5E,F), while the number of oospores generated by *PsBTS1* mutants also declined (Figures 5G,H). Specifically, the sporangia numbers of the four *PsBTS1* mutants decreased 38.4, 55.8, 36.9, and 34.8%, respectively, compared to the WT and CK (Figure 5F). Meanwhile, the oospore numbers of the mutants decreased by 49.8, 37.3, 40.7, and 39.2% (Figure 5H). The encystment rate of *PsBTS1* mutants was not affected (data not shown). To further examine the infectivity of *PsBTS1* mutants, a virulence assay was performed on susceptible soybean seedlings. The lesions on soybean seedlings inoculated with *PsBTS1* mutant zoospores were reduced in length (Figure 6A); the lesion lengths caused by inoculation with the four mutants were decreased by 60.6, 26.5, 22.7, and 51.5% compared to the WT (Figure 6B). The WT zoospores germinated and penetrated into epidermal cells on the inoculation sites at 12 and 24 hpi, respectively, while the zoospores of the *PsBTS1* mutants did not germinate to cause infection (Figure 6C). Such results indicate that PsBTS1 plays important roles during asexual and sexual reproduction as well as in the virulence of *P. sojae*.

### Gene Transcription Analysis

**Figure 7:**
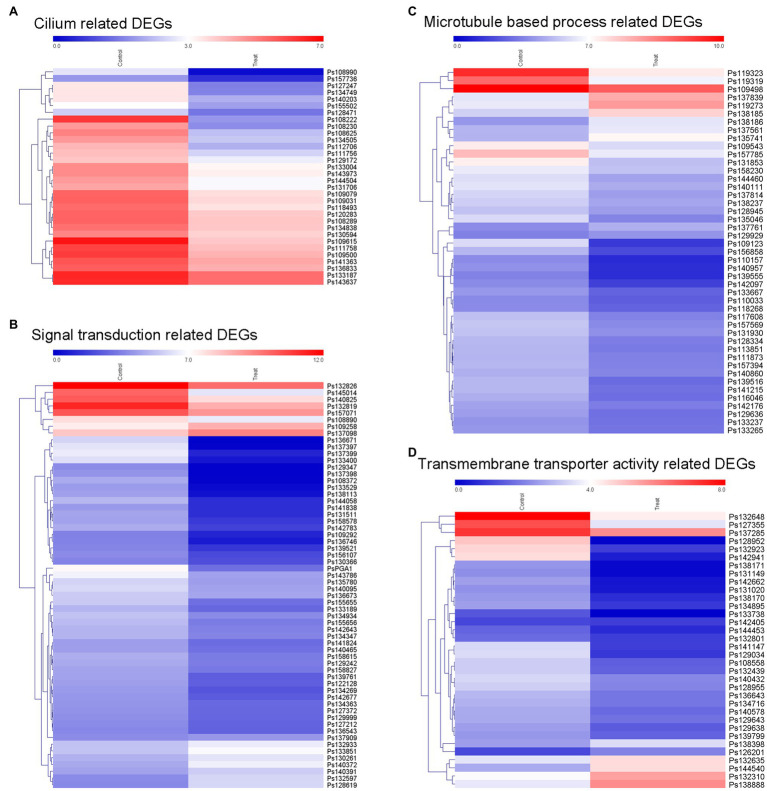
Expression of different groups of genes associated with sporangia formation under lovastatin treatment. The GO terms of DEGs are associated with signal transduction, assembly of zoospore structures, and transport of necessary components. The expression changes of different groups of **(A)** cilium-related genes, **(B)** signal transduction-related genes, **(C)** microtubule-based process-related genes, and **(D)** transmembrane transporter activity-related genes were clustered and visualized in a heatmap.

According to our results, the blocking of MVA pathway by lovastatin treatment or deletion of *PsBTS1* caused to reduced sporangia formation of *P. sojae*. Specifically, sporangia formation was significantly inhibited by lovastatin treatment (e.g., the concentration of 80 μg/ml) in this study. Such results suggest that sporangia formation is potentially related to the MVA pathway. To better elucidate the molecular mechanisms by which the MVA pathway mediates sporangia formation, transcriptome analysis was conducted by RNA-seq with *P. sojae* sporangia sample treated by lovastatin. In total, 4,018 differentially expressed genes (DEGs) were identified with a cut-off threshold of >2-fold change (*p* < 0.05), among which 1,393 genes were upregulated and 2,625 were downregulated (Supplementary Figure S7A). To predict the potential functions of the DEGs, Gene Ontology (GO) analysis was performed. The DEGs were primarily classified into biological process, cellular component, and molecular function (Supplementary Figure S7B). Specifically, the TOP 20 enriched GO terms (biological process) included cilium, signal transduction, microtubule-based process, and transmembrane transporter activity (Supplementary Figure S7C). The dynamic expression changes in key factors were revealed by the expression heatmap (Figure 7). The formation of sporangia involves complex regulation that requires signal transduction. In this study, all 37 identified cilium-related genes were downregulated (Figure 7A) and a total of 50 signaling pathway-related genes were identified with significant downregulation (Figure 7B). Microtubule-based process-related genes showed significant regulation, among which, 34 genes that were downregulated and 11 that were upregulated (Figure 7C). In total, 34 transmembrane transporter activity-related genes were detected, of which 28 were downregulated and six were upregulated (Figure 7D). In particular, dramatic expression changes in certain genes involved in signaling and cilia were identified, including the G protein alpha subunit (PsGPA1 and Ps108814) and phosphatase protein (PsCDC14 and Ps108222) in *P. sojae*, both of which were downregulated. Furthermore, the relative expression levels of *PsGPA1*, *PsCDC14*, and another six genes (*Ps158578*, *Ps142783*, *Ps109123*, *Ps139555*, *Ps109615*, and *Ps140432*) were validated by qRT-PCR in the lovastatin-treated sample and the representative KO mutant T4, all of which were downregulated, corroborating the RNA-seq results (Supplementary Figures S8A,B). The genes identified by RNA-seq analysis provide more information on the potential regulation of the MVA pathway in *P. sojae*.

## Discussion

Genome analysis indicated that many genes in sterol biosynthesis sub-branch of the MVA pathway are absent in *Phytophthora* spp., resulting in a sterol-auxotrophic life style (Tyler et al., 2006; Dahlin et al., 2017). Comparative analysis of sterol acquisition in sterol-auxotrophic and sterol-autotrophic oomycetes identified partial MVA pathway genes and the sterol biosynthesis sub-branch in *A. euteiches* and *S. parasitica* (Madoui et al., 2009; Dahlin et al., 2017). However, the complete MVA pathway in oomycetes has been little studied and its roles in development and virulence of these pathogens remain unknown. In this study, we blocked the MVA pathway by the enzyme inhibitor lovastatin which caused impaired growth, reproduction, and zoospore behavior of *P. sojae*. Subsequently, we identified a complete set of genes encoding enzymes that function in the MVA pathway in *P. sojae* and their homologues in related oomycete species. The enzyme inhibitor results and genes strongly suggest that the MVA pathway is ubiquitously conserved and functional in *Phytophthora* species. Deletion of the gene encoding the branch point enzyme PsBTS1 in *P. sojae* resulted in adverse effects on vegetative growth, reproductive development, and virulence similar to those caused by lovastatin treatment. Transcriptome analysis also further indicated that the MVA pathway potentially regulates growth, reproduction, and virulence characters in *P. sojae*.

The MVA pathway has been identified and is evolutionarily conserved in animals, plants, and fungi (Dimster-Denk et al., 1994; Fabris et al., 2014; Ruiz-Sola et al., 2016). The product of the initial steps in the MVA pathway, i.e., farnesyl diphosphate (FPP), feeds into different sub-branches to produce isoprenoids, sterols, ubiquinone, and dolichols, respectively (Fabris et al., 2014; Athanasakoglou et al., 2019). As an important metabolic pathway in cholesterol biosynthesis, the MVA pathway has been excavated as the pharmacological target of statins (Thompson and Taylor, 2017). The enzyme inhibitor statin targeted the rate-limiting enzyme HMGR to decrease cholesterol level in serum for hypercholesterolemia (Istvan and Deisenhofer, 2001; Stancu and Sima, 2001). The protein sequence, and the characteristics of the functional and binding domains in fungal HMGR share high similarity with the human homologue, which implies that statins also inhibit HMGR in fungal species (Bochar et al., 1999). Different formulations of statin were consistently found to inhibit growth of *Candida* species and *Aspergillus fumigatus* effectively (Macreadie et al., 2006; Westermeyer and Macreadie, 2007). In addition to the observation that certain statins act as growth inhibitors, statins are also found to reduce biofilm production in the human pathogenic fungus *Candida albicans* (Liu et al., 2009), to increase the frequency of petite cells in *Candida glabrata* (Westermeyer and Macreadie, 2007), and to decrease germination in *Rhizopus oryzae* and cause melanin loss, reduced virulence, and increased susceptibility to oxidative stress in this species (Bellanger et al., 2016). Moreover, a decrease in ergosterol was confirmed in *Candida* species and *A. fumigatus* treated with statins, while treatments supplemented with ergosterol or cholesterol rescued statin-induced growth inhibition (Macreadie et al., 2006). These results indicated the similar effects of statins in sterol biosynthesis in human and fungi, the growth inhibition in fungi caused by statins is related to decreased sterol (Westermeyer and Macreadie, 2007; Bellanger et al., 2016).

Lovastatin treatment impeded development and virulence of *P. sojae*, which indicates a functional MVA pathway in *P. sojae*. *Phytophthora* zoospores are specific structure for short distance dispersal, which swim for hours with the help of flagella (Judelson and Blanco, 2005) and thereafter encyst on host epidermal cells to cause infection (Hua et al., 2008). Therefore, lovastatin was reported to impede zoospores dispersal due to the increase of encystment rate in this study. Moreover, the complete set of MVA pathway genes were found to be present in the genome of *P. sojae*, and all nine genes were found to be expressed. The different expression of these genes was supposed to play potential function in certain developmental or infection stages of *P. sojae*. Moreover, the homologues in the MVA pathway in *P. sojae* have also been identified in other *Phytophthora* species, which further indicates that the MVA pathway is conserved in *Phytophthora* species. However, the effects of statin as anti-*Phytophthora* agent were probably not identical to those in fungi because *Phytophthora* species specially lack sterol biosynthesis pathway (Tyler et al., 2006; Dahlin et al., 2017). The GGPS is a branch point enzyme in the terpenoid biosynthesis sub-branch of the MVA pathway (Alcaino et al., 2014). GGPSs are classified into three types based on the aspartate-rich motifs (Beck et al., 2013; Kato et al., 2016). The phylogenetic analysis revealed that oomycetes were closely related to two diatoms. This result is consistent with the fact that oomycetes and diatoms fall within the kingdom stramenopila and share a common ancestor (Tyler et al., 2006). Numerous GGPSs have been identified in plants and fungi that were found to localize in the cytoplasm or different organelles (Okada et al., 2000; Saikia and Scott, 2009; Beck et al., 2013). Thereby, the subcellular localization will suggest sub-functionalization by providing GGPP to specific tissues and even developmental stages (Beck et al., 2013; Coman et al., 2014). In plants, GGPS modulates development and growth by regulating biosynthesis of photosynthetic pigments and plant hormones (Ruppel et al., 2013; Ruiz-Sola et al., 2016; Tata et al., 2016; Zhou et al., 2017). For example, GGPSs in *A. thaliana* and *Oryza sativa* are involved in chlorophyll biosynthesis, and disruption leads to dwarf and leaf chlorosis phenotypes (Ruppel et al., 2013; Ruiz-Sola et al., 2016; Zhou et al., 2017).

Fungal GGPSs are involved in synthesis of diterpenes, such as crtE regulating astaxanthin production in *Xanthophyllomyces dendrorhous* (Alcaino et al., 2014), ggs2 affecting helvolic acid production in *M. anisopliae* (Singkaravanit et al., 2010a), and paxG associated with paxilline biosynthesis in *Penicillium paxilli* (Saikia and Scott, 2009). However, functional studies of GGPP in fungi are rare, with a few examples, such as deletion of the GGPS resulting in slow growth of *S. cerevisiae* under cold stress (Jiang et al., 1995) and deficiency of *ggs2* in *M. anisopliae* causing delayed sporulation and weak toxicity with no clear underlying mechanisms (Singkaravanit et al., 2010a). In our study, *PsBTS1* was identified and its encoded protein with enzymatic activity was localized in the cytoplasm. In addition, the monoallelic editing of *INF1* gene in *P. infestans* had been reported using CRISPR/Cas12a system recently, in which a copia-like element in the promoter region of *INF1* was inserted and presumably impeded the allele gene editing (Ah-Fong et al., 2021). According to current results in this study, *PsBTS1* editing resulted in defective mycelial growth, reduced sporangia and oospore numbers, and delayed germination of cysts on host surfaces, leading to decreased virulence in *P. sojae*. These phenomena were similar to the effects of lovastatin treatment. These results indicate the essential roles of the MVA pathway during the development, reproduction, and virulence of *P. sojae*.

Transitions between different stages in the life cycle contribute to the infection success of plant pathogenic oomycetes (Judelson and Blanco, 2005). Previous studies investigated transcriptional changes during the life cycles of *P. infestans* and *P. sojae* (Judelson et al., 2008, 2009; Ye et al., 2011). In particular, calcium-binding proteins, flagellar proteins, signaling proteins, and cation channel-encoding genes are upregulated at the sporangia stage (Judelson et al., 2008; Ah-Fong et al., 2017). In our study, sporangia formation was inhibited and sporangium-related gene expression was highly suppressed. For example, downregulated expression of genes encoding PsGPA1 and PsCDC14 was observed in this study. GPA1 regulates zoospore motility and virulence in *P. sojae* (Hua et al., 2008) and *P. infestans* (Latijnhouwers et al., 2004). CDC14 is expressed mainly in sporangia and regulates sporangia formation in *P. infestans* (Ah-Fong and Judelson, 2003, 2011). We hypothesize that the MVA pathway regulates growth, reproduction, and virulence by orchestrating dynamic changes at the transcriptome level in *P. sojae*.

In this study, we identified the complete MVA pathway in *P. sojae* and revealed its ubiquity in *Phytophthora* species. We demonstrated that the MVA pathway regulates growth, reproduction, and virulence in *P. sojae* through non-sterol pathway. Certain genes related to development and virulence were identified as being potentially affected by the MVA pathway in *P. sojae*. This research provides new clues about the molecular mechanisms involved in regulating development and virulence in *P. sojae*, which differ from those in fungi. Our findings may assist in the development of sustainable disease-management strategies and chemical targets for diseases caused by *P. sojae* and other *Phytophthora* species.

## Data Availability Statement

The original contributions presented in the study are publicly available. This data can be found here: PRJNA767514.

## Author Contributions

XY: conceptualization, formal analysis, investigation, writing – original draft, writing-review and editing, supervision, and funding acquisition. XJ, WYa, and QH: formal analysis, investigation, and visualization. HS, XZ, and ZZ: investigation. WYe and YW: formal analysis. FG: writing-review and editing. YL: conceptualization and writing-review and editing. All authors contributed to the article and approved the submitted version.

## Funding

This work is supported by the National Natural Science Foundation of China (grant nos: 31601586 and 31601615), the Scientific Research Foundation of Educational Department of Liaoning Province (LSNQN202015) and Starting Grants of Shenyang Agricultural University (880416060).

## Conflict of Interest

The authors declare that the research was conducted in the absence of any commercial or financial relationships that could be construed as a potential conflict of interest.

## Publisher’s Note

All claims expressed in this article are solely those of the authors and do not necessarily represent those of their affiliated organizations, or those of the publisher, the editors and the reviewers. Any product that may be evaluated in this article, or claim that may be made by its manufacturer, is not guaranteed or endorsed by the publisher.

## Footnotes

^1^ https://www.yeastgenome.org/

^2^ https://jgi.doe.gov/
