# Supplementary material for: The Mevalonate Pathway Is Important for Growth, Spore Production, and the Virulence of Phytophthora sojae
Source: Front Microbiol. 2021 Dec 22;12:772994. doi: 10.3389/fmicb.2021.772994 (PMC9635365; doi:10.3389/fmicb.2021.772994)
Supplement: Supplementary file 1 [file Data_Sheet_1.docx]

**Supplementary information**

**Supplementary Figure S1∣**Lovastatin suppresses growth of different oomycete species. Colonies and growth rates of **(A, B)** *P. infestans*, **(C, D)** *P. capsici*, and **(E, F)** *Py. ultimum* under treatment with various concentrations of lovastatin. Different concentrations (0, 20, 40, and 80 μg/ml) of lovastatin were added to the plates. The colonies were record and growth rates were calculated; a, b, and c indicate significant differences (*p* < 0.05).

**Supplementary Figure S2∣**Expression patterns of MVA pathway genes in different development and infection stages of *P. sojae*. The expression data of putative MVA pathway genes from *P. sojae* were used to generate a heatmap using Multiple Experiment Viewer. MY: mycelium, SP: sporangia, ZO: zoospores, CY: cysts, GC: germinated cysts and hours post inoculation (hpi) of zoospores to soybean roots (1.5, 3, 6, 12 and 24 h).

**Supplementary Figure S3∣**Alignment of conserved motifs in GGPSs from taxonomically different organisms. GGPSs were classified into three types based on the first aspartate-rich motif (FARM) and the second aspartate-rich motif (SARM); the conserved FARM, SARM, and G(Q/E) motifs are indicated by red boxes.

**Supplementary Figure S4∣**Subcellular localization of GFP signal in mycelial. The GFP signal in mycelial of PsBTS1-GFP and GFP expression *P. sojae* were analyzed by fluorescence (GFP, 488/515 nm). Bar: 5 μm.

**Supplementary Figure S5∣**CRISPR/Cas9-mediated gene deletion of *PsBTS1*. **(A).** Strategy of gene deletion mediated by CRISPR/Cas9 and location of the primers used to screen the deletion mutants. Red triangle indicated target sites of sgRNA. **(B)**. Screen of *PsBTS1* edited mutants (T4, T23, T32, and T50) by 5’ junction PCR (primer pair F2/R2), 3’ junction PCR (primer pair F3/R3), spanning PCR (primer pair F2/R3) and internal PCR (primer pair F1/R1). **(C)**. Relative expression levels of *PsBTS1* in four mutants. Asterisk indicated significant differences. **(D)**. Relative expression levels of two neighbor genes (*Ps127815* and *Ps127817*) adjacent to *PsBTS1* in mutant T4 and T23. NS: no significant.

**Supplementary Figure S6∣**The distance between mycelial branches (DBMB) of PsBTS1 deletion mutants, WT and CK strains. The *PsBTS1* KO mutants exhibit rough and irregular colony edges where small satellite mycelial colonies are formed. The DBMB values were compared between WT, CK, and *PsBTS1* KO mutants T4, T23, T32, and T50. Asterisk indicated significant differences.

**Supplementary Figure S7∣**Transcriptome analysis of DEGs under lovastatin treatment. **(A)**. DEGs under treatment with lovastatin during sporangium formation. Genes were considered differentially expressed with a fold change ≥ 2 and a p-value ≤ 0.05. The x coordinate represents log2 values of fold change; the y coordinate represents −log10 values of p-value. **(B)**. GO classification analysis of DEGs. The x coordinate represents the number of genes; the y coordinate represents the biological processes. **(C)**. GO enrichment analysis of DEGs. The x coordinate represents the rich ratio of genes; the y coordinate represents the enriched biological processes; the color of the bar represents the corrected p-value size.

**Supplementary Figure S8∣**Quantitative reverse transcription PCR verification of DEGs in lovastatin-treated samples and a PsBTS1-deletion mutant. The relative expression levels of eight DEGs related to the signaling pathway related genes (*PsGPA1*, *Ps158578*, *Ps142783*), microtubule formation related genes (*Ps109123*, *Ps139555*), cilium formation related genes (*PsCDC14*, *Ps109615*), and movement of cell component related gene (*Ps140432*) were verified in the **(A)** lovastatin-treated samples and **(B)** *PsBTS1*-deletion mutant T4 during the sporangia-formation stage. Asterisk indicated significant differences.

**Supplementary Table S1∣**The sequence information of proteins in MVA pathway in *P. sojae*. The proteins information in MVA pathway of *P. sojae* were listed in file. The gene ID are based on Joint Genome Institute database and the accession number are based on GenBank. All the gene model was checked and two genes (Ps109674, Ps156915) were manually annotated based on our RNA-seq data at the first time.

**Supplementary Table S2∣**Prediction of MVA pathway proteins in *P. sojae* and other organisms. The MVA pathway proteins of *P. sojae* (Joint Genome Institute Database, JGI) are indicated in the first column. The proteins in MVA pathway of *P. sojae* were used to search homologues in *S. cerevisiae*, *P. infestans*, *S. parasitica*, *P. capsica, Py. ultimum* using the BLASTP program. The ID or accession number of the homologues and the protein sequence identity were listed for different organisms. The identified homologues from the Uniprot database with higher identities were listed in the last column.

**Supplementary Table S3∣**Cilium-related differentially expressed genes.

**Supplementary Table S4∣**Signal transduction related differentially expressed genes.

**Supplementary Table S5∣**Microtube based process related differentially expressed genes.

**Supplementary Table S6∣**Transmembrane transporter activity related differentially expressed genes.

**Supplementary Table S7∣**Primers used in this study.
