# Supplementary figures and images for: The Mevalonate Pathway Is Important for Growth, Spore Production, and the Virulence of Phytophthora sojae
Source: Front Microbiol. 2021 Dec 22;12:772994. doi: 10.3389/fmicb.2021.772994 (PMC9635365; doi:10.3389/fmicb.2021.772994)

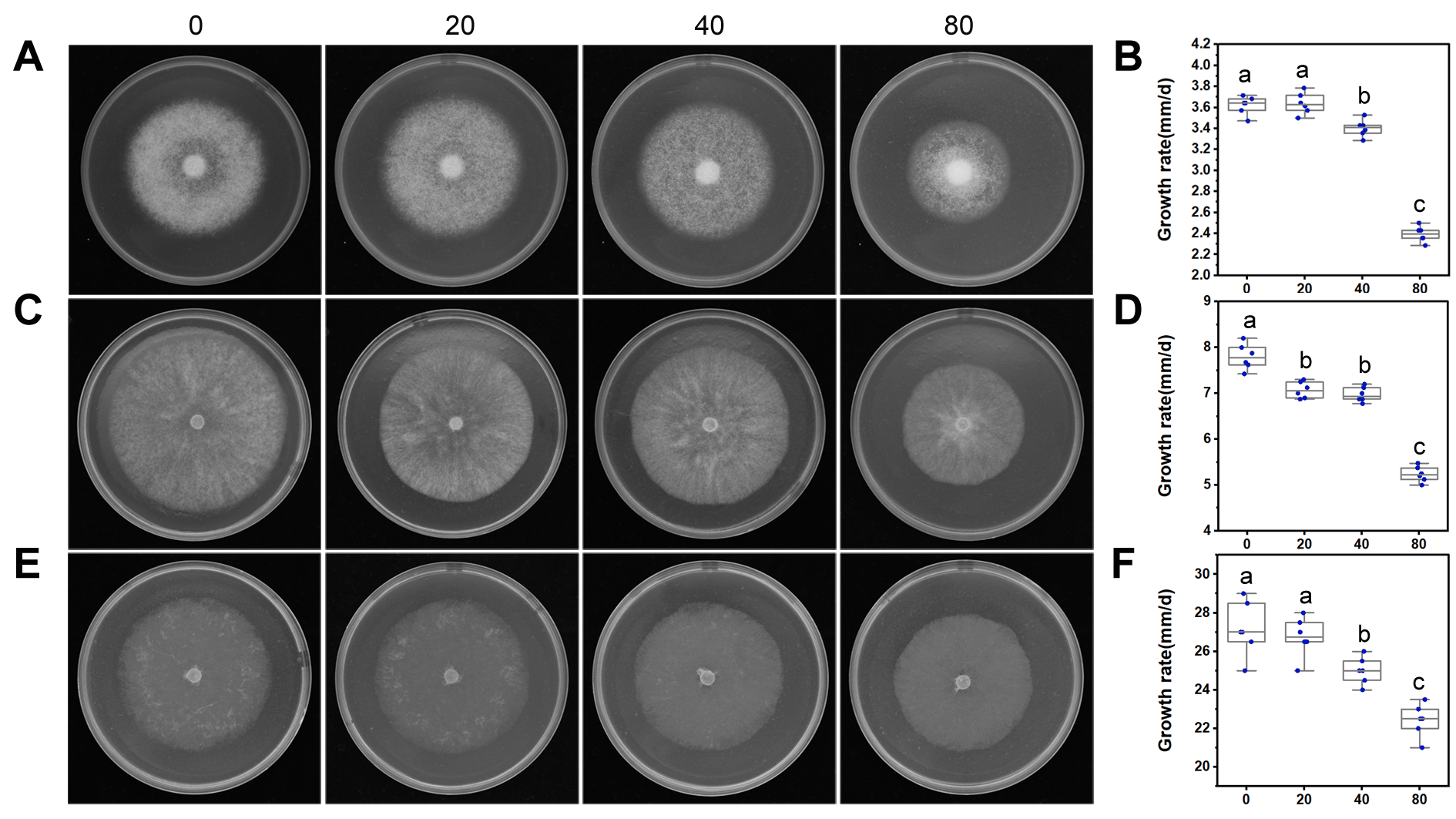

Supplement: Supplementary file 3 [file Image_1.TIF]

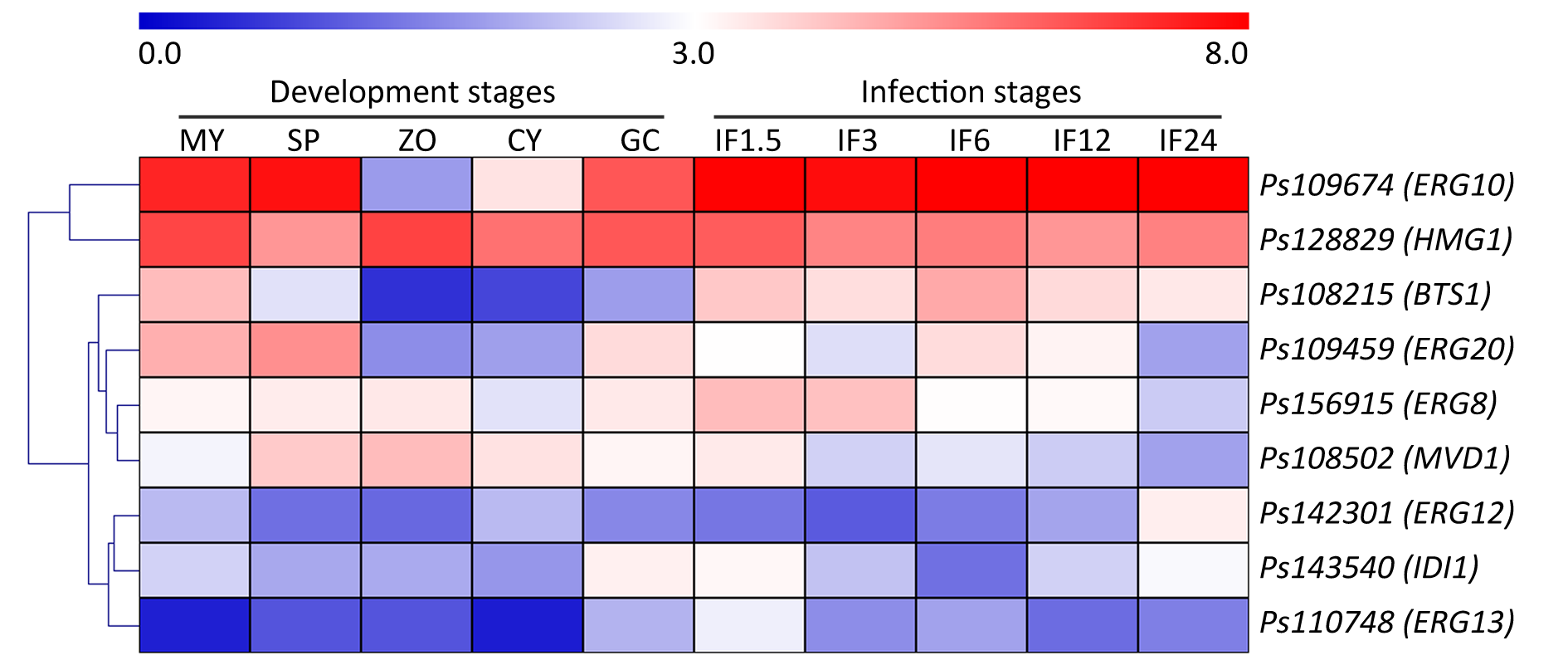

Supplement: Supplementary file 4 [file Image_2.TIF]

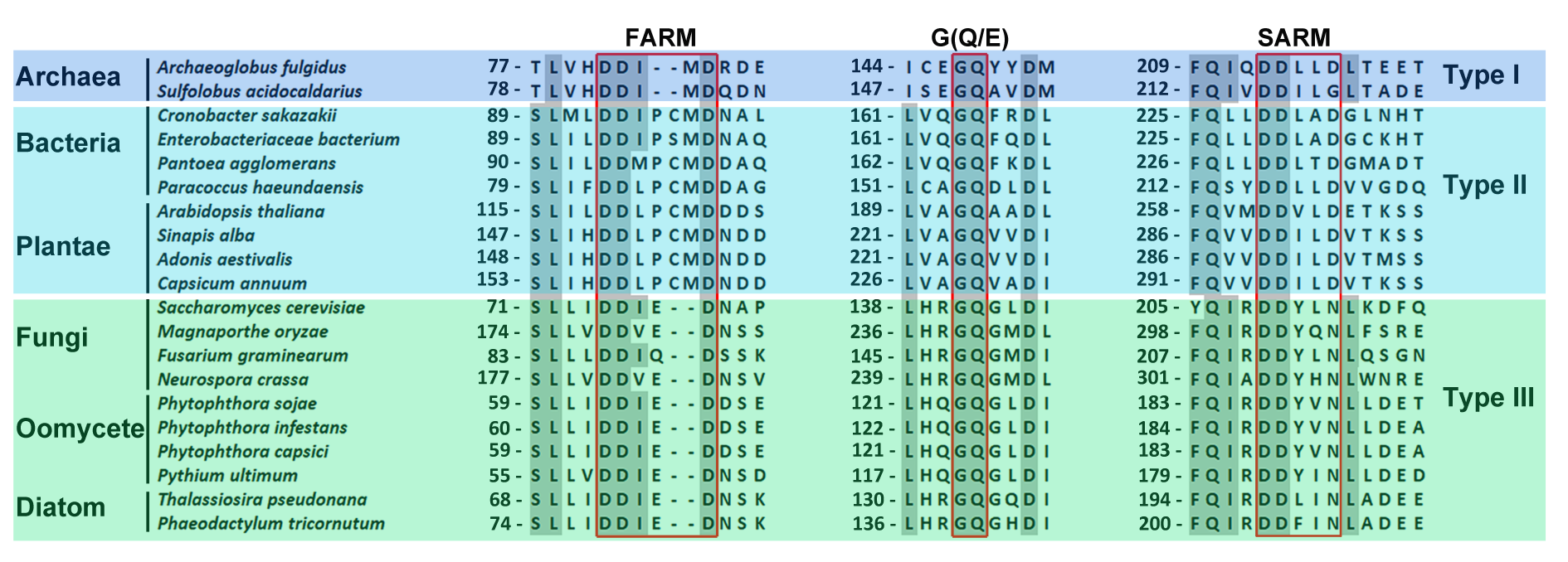

Supplement: Supplementary file 5 [file Image_3.TIF]

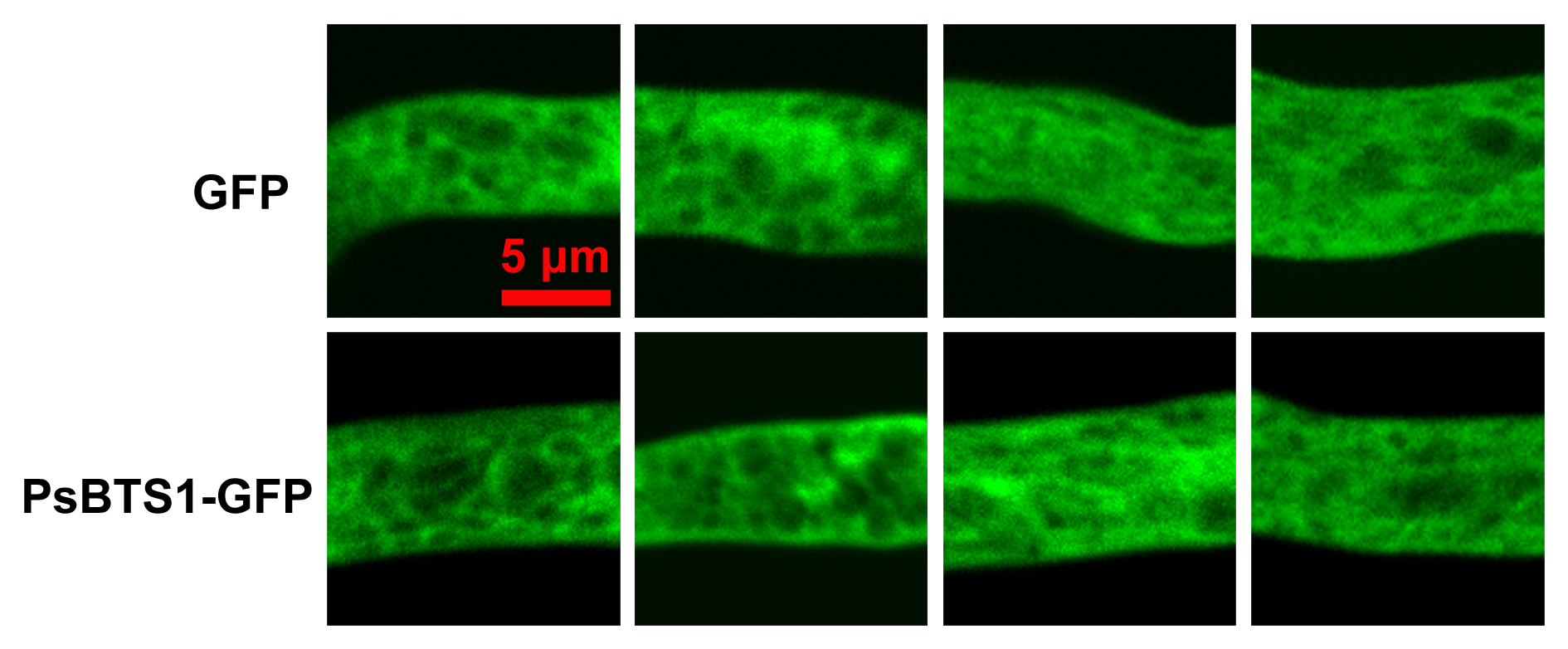

Supplement: Supplementary file 6 [file Image_4.TIF]

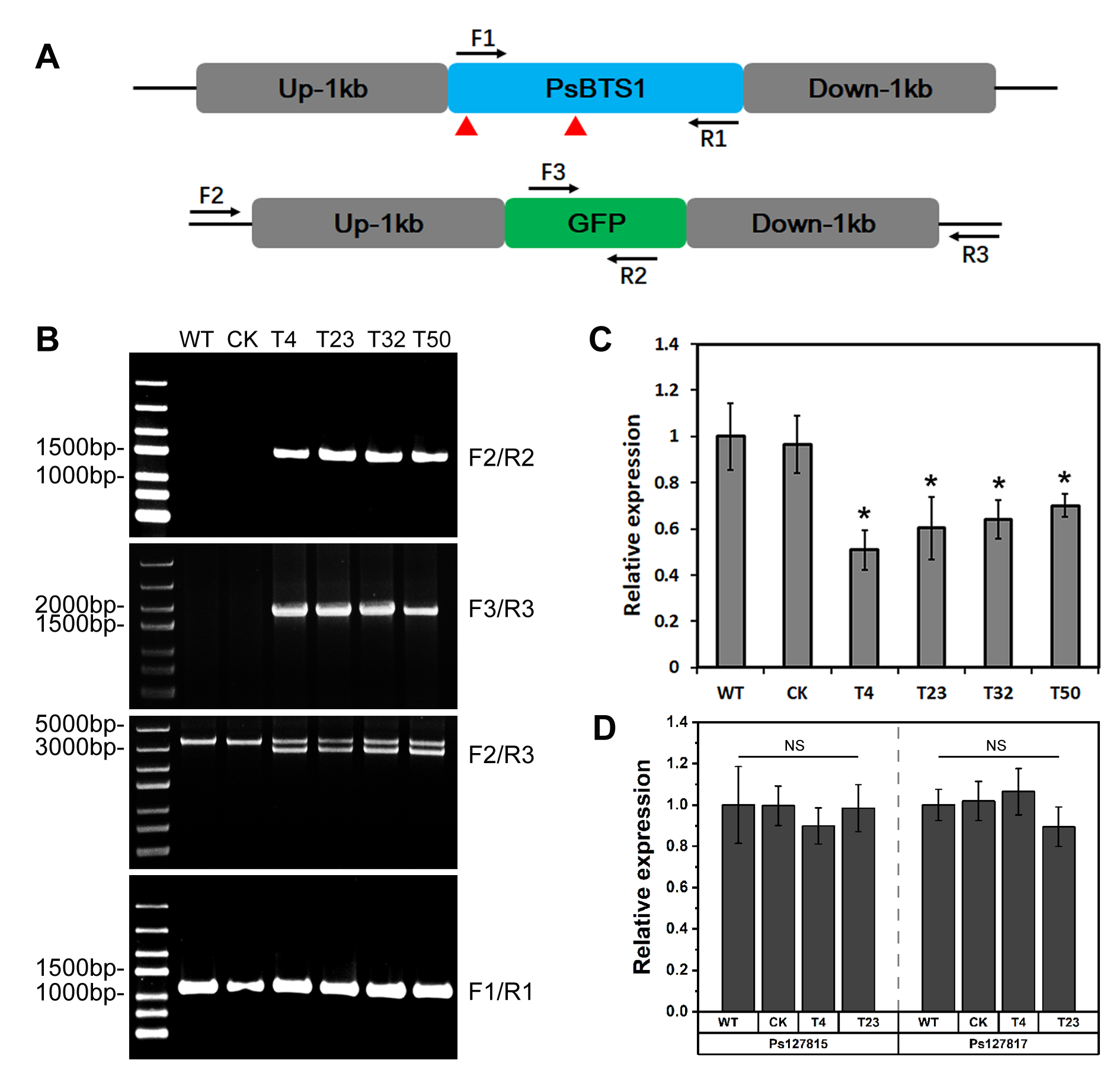

Supplement: Supplementary file 7 [file Image_5.TIF]

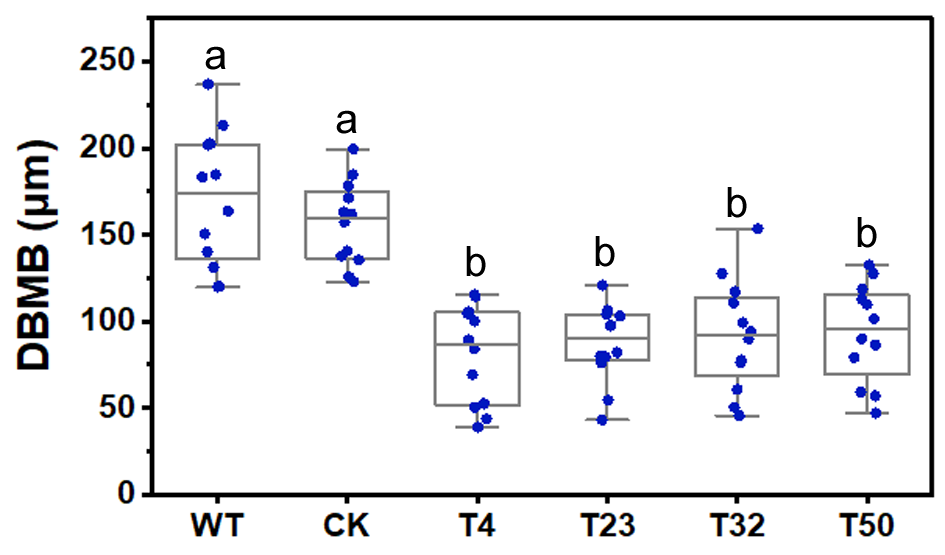

Supplement: Supplementary file 8 [file Image_6.TIF]

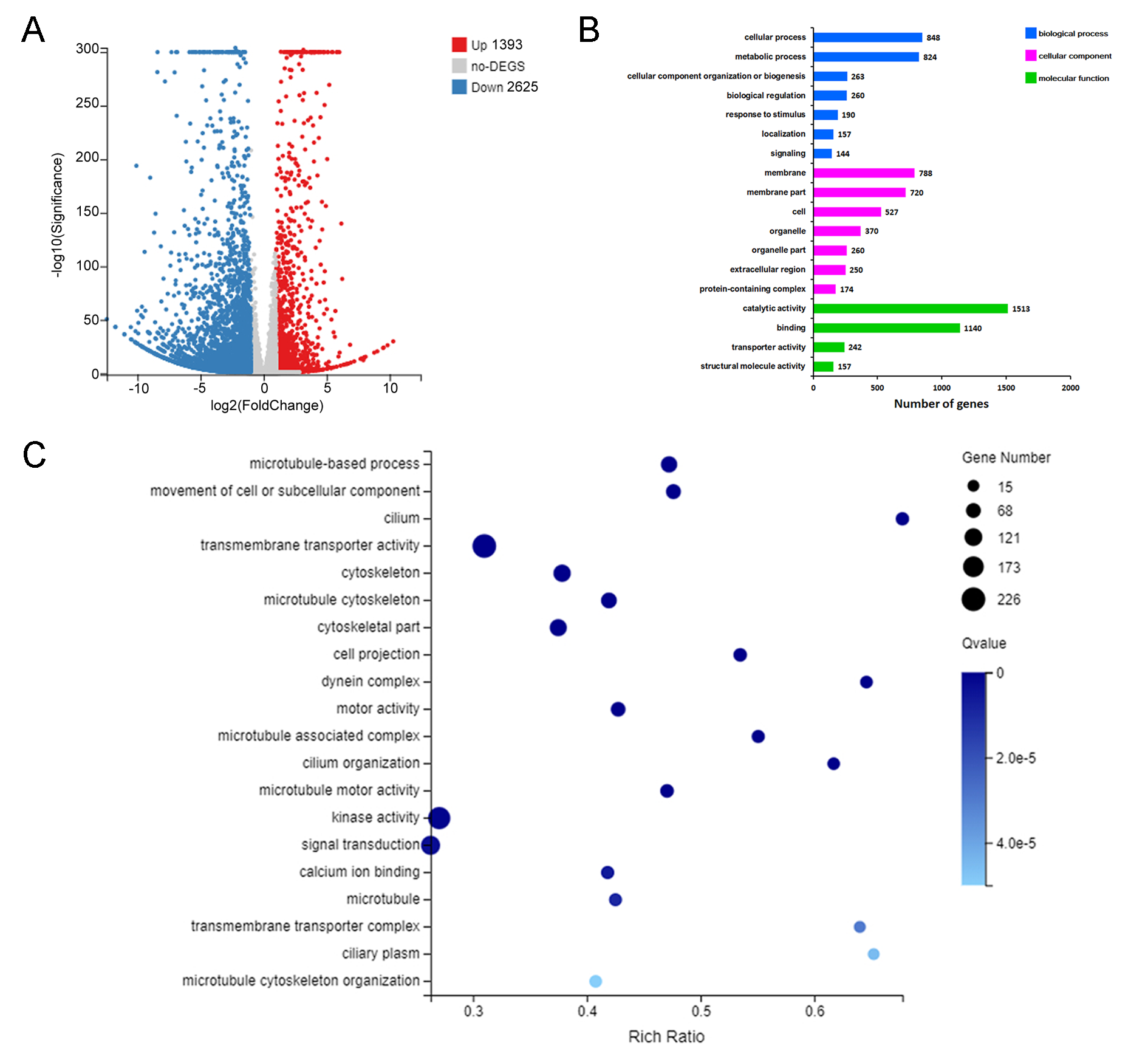

Supplement: Supplementary file 9 [file Image_7.TIF]

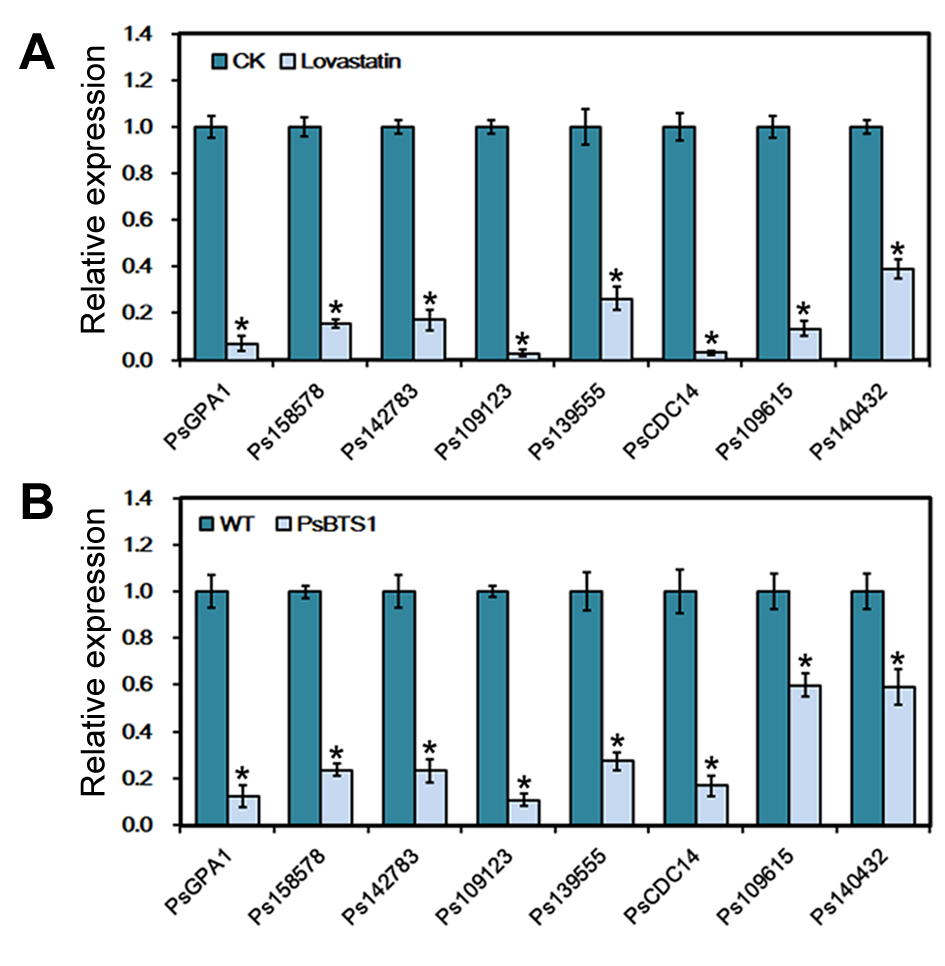

Supplement: Supplementary file 10 [file Image_8.TIF]
